# Supplementary material for: Aluminum Thin Film Nanostructure Traces in Pediatric EEG Net for MRI and CT Artifact Reduction
Source: Sensors (Basel). 2023 Mar 31;23(7):3633. doi: 10.3390/s23073633 (PMC10098641; doi:10.3390/s23073633)
Supplement: Supplementary file 1 [file sensors-23-03633-s001.zip › sensors-2280871-supplementary.pdf]

Supplement: Aluminum Thin-Film Nanostructure Traces in Pediatric EEG Net for MRI and CT Artifact Reduction

Hongbae Jeong [1,†], Georgios Ntolkeras [2,3,†], Tracy Warbrick [4], Manfred Jaschke [4], Rajiv Gupta [5], Michael H. Lev [5], Jurriaan M. Peters [6], Patricia Ellen Grant [2] and Giorgio Bonmassar [1,2,*]

[1] AA. Martinos Center, Massachusetts General Hospital, Harvard Medical School, Charlestown, MA 02129, USA
[2] Department of Newborn Medicine, Fetal-Neonatal Neuroimaging and Developmental Science Center, Boston Children's Hospital, Boston, MA 02115, USA
[3] Department of Pediatrics, Baystate Medical Center, University of Massachusetts Medical School, Springfield, MA 01605, USA
[4] Brain Products GmbH, 82205 Gilching, Germany
[5] Department of Radiology, Massachusetts General Hospital, Harvard Medical School, Boston, MA 02114, USA
[6] Department of Neurology, Boston Children's Hospital, Harvard Medical School, Boston, MA 02115, USA
* Correspondence: giorgio.bonmassar@mgh.harvard.edu; Tel.: +1-617-726-0962
† These authors contributed equally to this work.

S.1 Clinical Rationale for the development of the NeoNet.

Central nervous system disorders in neonates may present as encephalopathies and are often accompanied by seizures. Acute symptomatic seizures in neonates may stem from perinatal hypoxic-ischemic injury, or stroke, but epilepsy in neonates can also stem from genetic-metabolic etiologies or brain structural abnormalities. Magnetic Resonance Imaging (MRI) plays a crucial role in diagnosing and understanding the cause of neonatal seizures. However, neonatal MRI evaluation assesses only structure, not function [1]. In such circumstances, continuous video EEG provides essential information about epileptiform abnormalities and seizures (frequency, distribution, and location), and the overall brain function is reflected in the background patterns. The latter evolves with gestational age and has age-specific norms describing typical grapho-elements and matural background patterns in awake and sleep states. In critically ill neonates, infants, and young children in the ICU, EEG monitoring is the only widely accepted clinical tool that provides a continuous and direct window to brain function [2]. Finally, EEG can also inform clinicians about developmental brain patterns in children with developmental disabilities in early childhood. State-of-the-art EEG or dense array EEG (HD-EEG – 64 or more channels) has enabled the realization of EEG's potential as a neuroimaging tool through source localization of normal and pathological brain activity and network dynamics. The quandary is that EEG caps/electrodes must be removed before neuroimaging since EEG produces visible and significant artifacts that can compromise the CT and MRI quality (**Fig. 6, 7** in the main manuscript). However, such electrodes may produce significant image artifacts in MRI sequences such as SWI and DWI, which are sensitive to the changes in the magnetic susceptibility between the metals used in EEG and surrounding tissue. Radiologists will refuse to perform clinical evaluations of MRI images with EEG due to the risk of missing very subtle pathological changes (false negative) or misdiagnosing changes as pathological (false positive) due to B1 field distortions caused by the EEG leads [3, 4]. Furthermore, EEG leads raise significant safety issues with MRI (see **Introduction** in the main manuscript). Finally, applying, removing, and reapplying EEG electrodes causes significant delays in treatment and adds unnecessary clinical costs because (1) correct placement is time-consuming, (2) it disrupts the logistics and the workflow, and (3) it produces skin irritation since the skin needs preparation before EEG electrode application and once the skin is prepared it becomes sensitized, especially in neonates. For that reason, our team has developed a CT, MRI conditional EEG net, the NeoNet, that has enabled pediatric simultaneous EEG/MRI recordings.

EEG is widely used in the NICU and is the gold standard for monitoring/assessing brain function in preterm and term neonates. EEG is critical in the diagnosis of seizures, for monitoring the recovery from hypoxic-ischemic events, and in the assessment of brain maturity. In all of these situations, high-density EEG (HD-EEG) is preferred as it provides the best signal-to-noise ratio (SNR), dynamic range, and sampling rate compared to the older 10-20 EEG. Furthermore, HD-EEG has enhanced artifact removal and can detect seizures [5] automatically. For example, neonatal EEGs may display sharp focal transients or epileptic discharges that appear in only a single electrode (7-9). This transient may be lost if smoothed by volume conduction but recovered by spatially filtered (e.g., Laplacian [6-9]) HD-EEG. The advantage of HD-EEG is also well-known in source localization [10], which has potential NICU applications in the presurgical evaluation of neonatal epilepsies due to cortical malformations [11]. Cortical resection or hemispherectomy can result in the cessation or dramatic reduction in the frequency of seizures in infants admitted to the NICU with severe, intractable epilepsy [12, 13]. HD-EEG is safe in the NICU, with the application of a proper EEG cap no more stressful to the neonate than a routine care procedure [14]. For these reasons, HD-EEG is most likely a desired technology in NICUs worldwide. HD-EEG also enables new clinical discoveries, such as regional maturational changes in neonatal brain connectivity [15]. In addition, in patients with epilepsy, the combination of advanced neurophysiologic and neuroimaging studies has allowed for a better understanding of different pathologies and facilitates treatment. For patients with drug-

resistant epilepsy, surgery is the only therapeutic option that can control or cure their seizures [16]. EEG-fMRI can help identify the seizure origin by performing advanced analysis on well-established biomarkers for epilepsy, such as interictal spikes [17]. In addition, incorporating EEG and fMRI has been shown to increase the sensitivity of the presurgical evaluation process and reveal new regions contributing to the epileptic phenotype of patients with focal epilepsy [18]. EEG-fMRI has also been shown to increase the number of surgical candidates, especially those with the challenging to localize focal epilepsy, reporting good postsurgical outcomes [19]. As a result, incorporating EEG and fMRI can significantly benefit patients who seek treatment for drug-resistant epilepsy, having a substantial role in the presurgical evaluation of those patients.

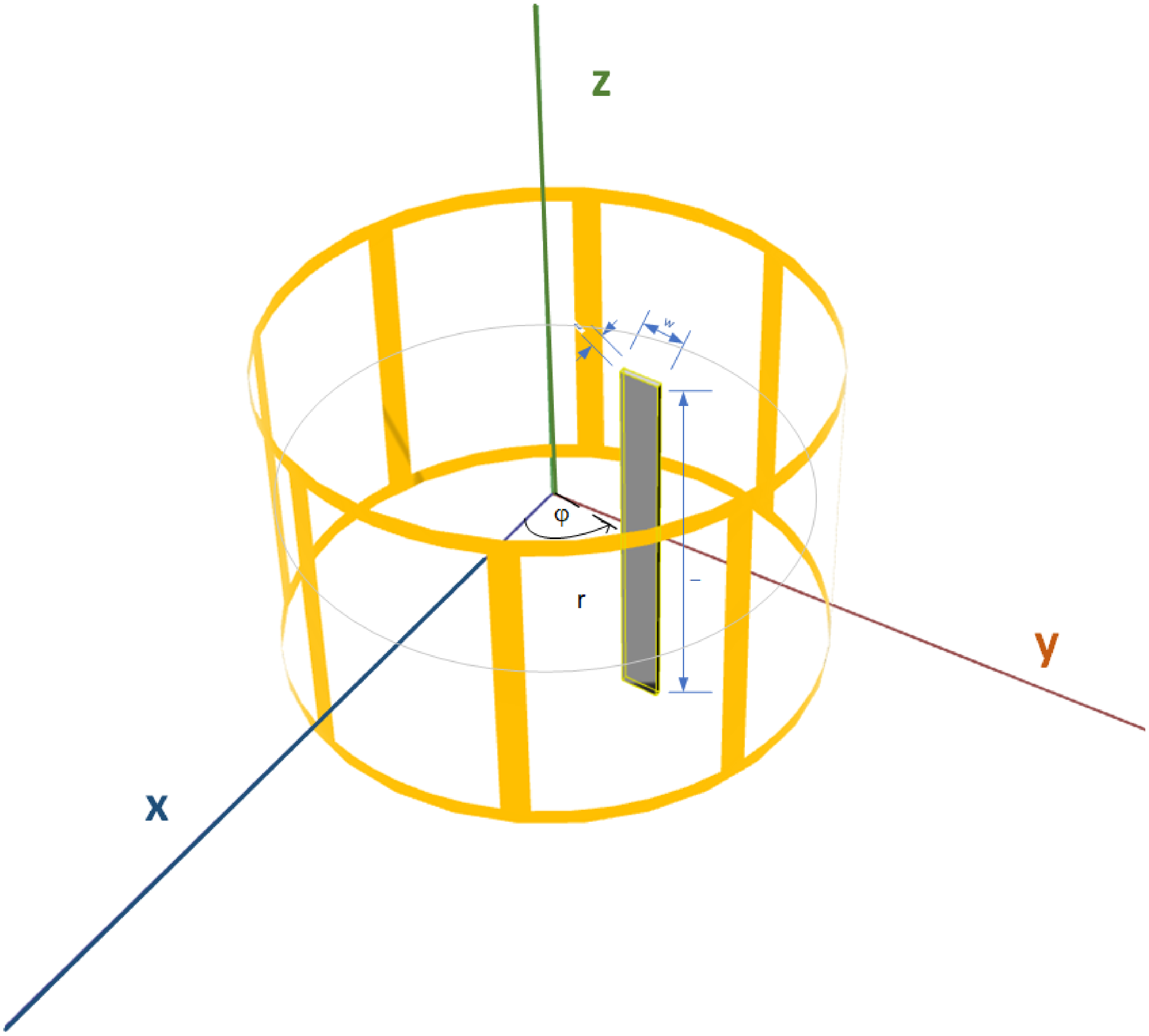

Figure S1: Geometrical model of the birdcage and thin film used in the Theory.

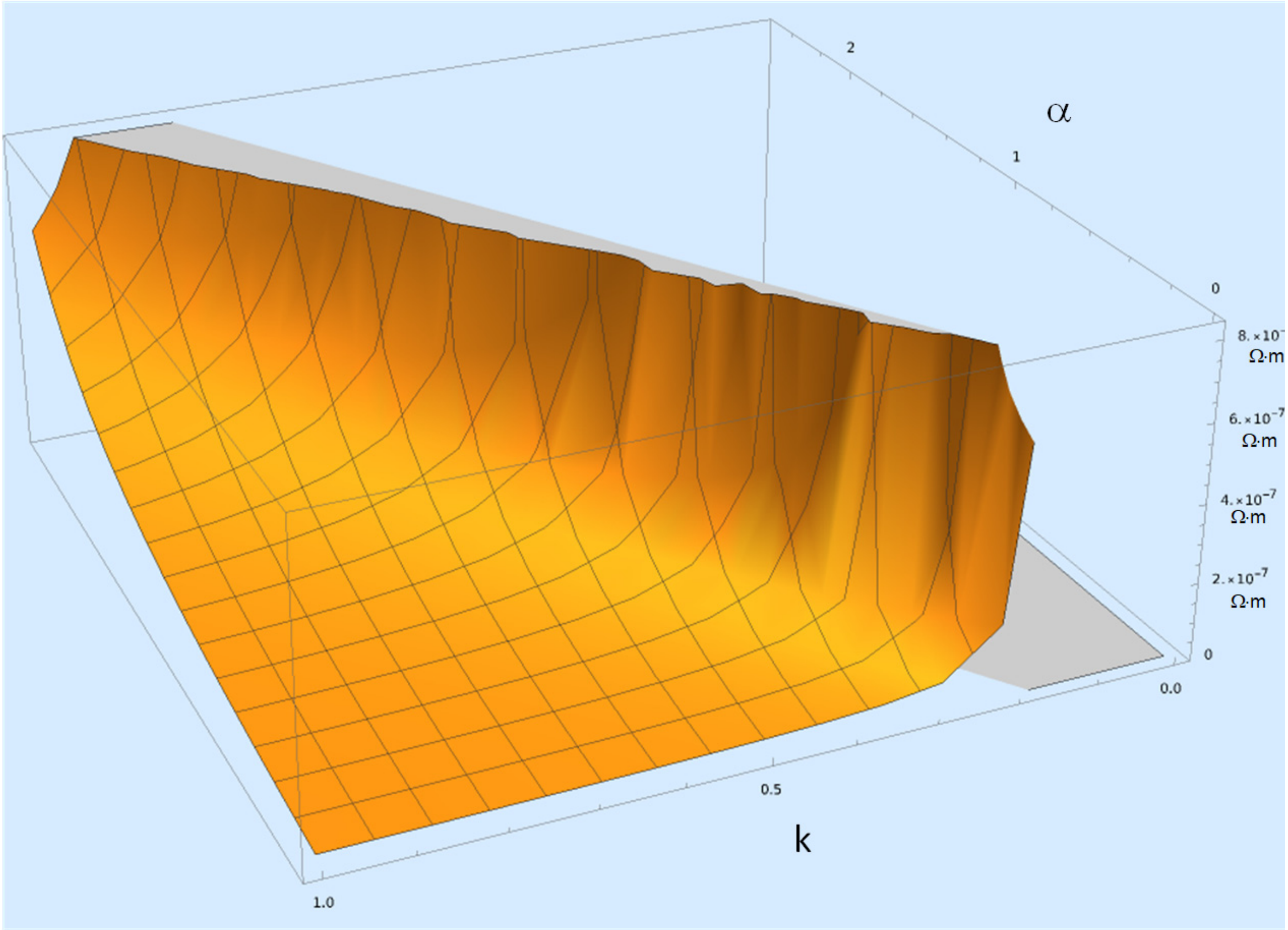

Figure S2: The resistivity according to the FS-MS model for Al thin films.

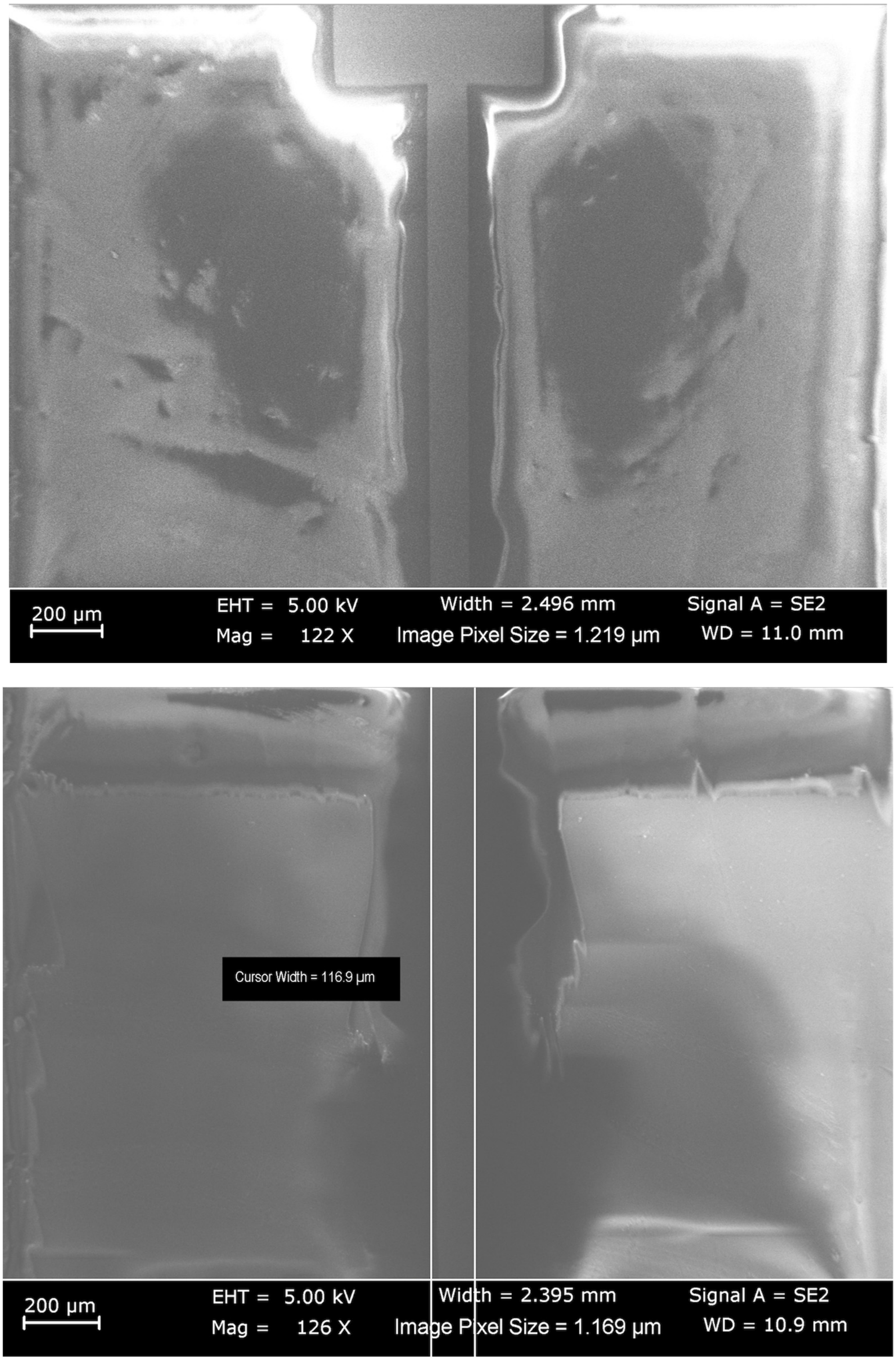

Figure S3: The NeoNet trace SEM images; *(Top) Detail of a trace and the pad of a connector; (Bottom) The measurement of the traces returned 117µm, which is within the 101.6 µm of the design value.*

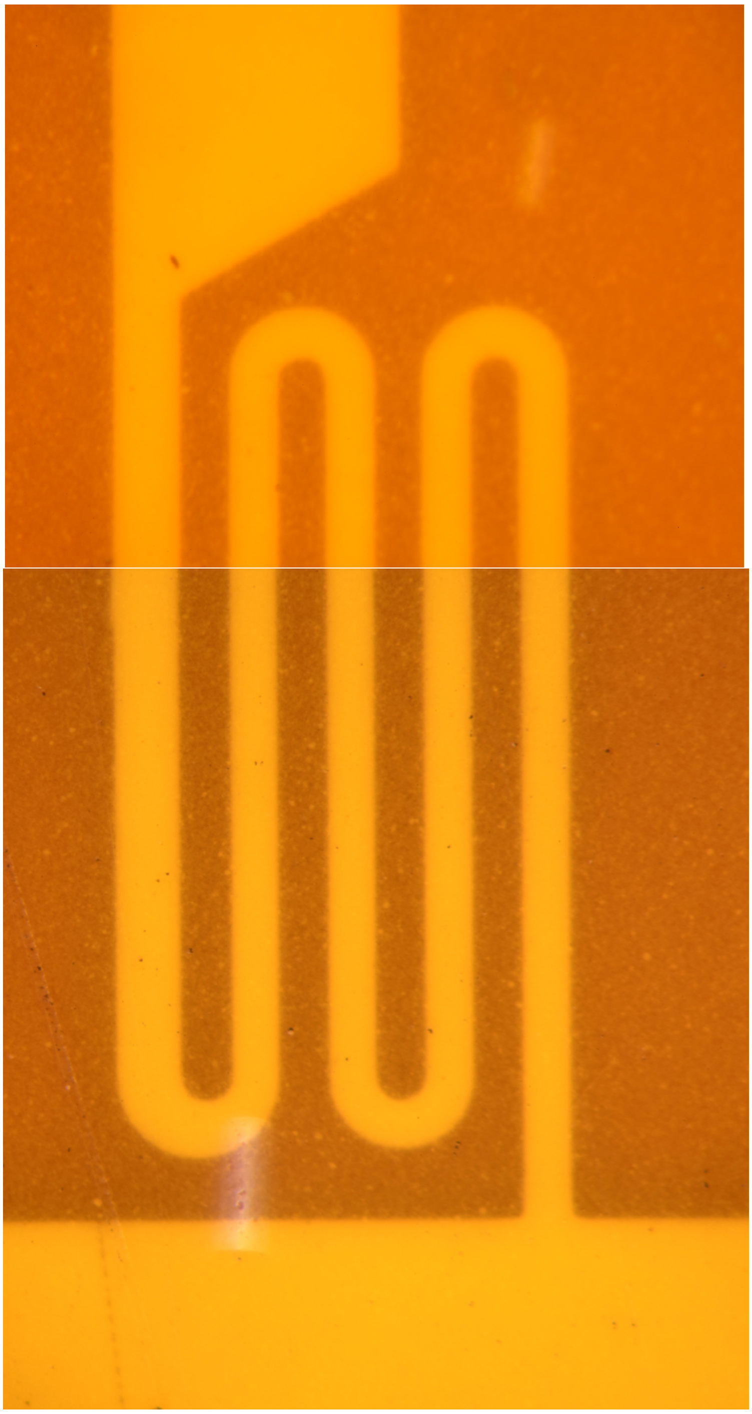

Figure S4: Microscope images of the Five traces loop. *(Top) Each electrode is connected to the pad; (bottom) through five long traces connected in series.*

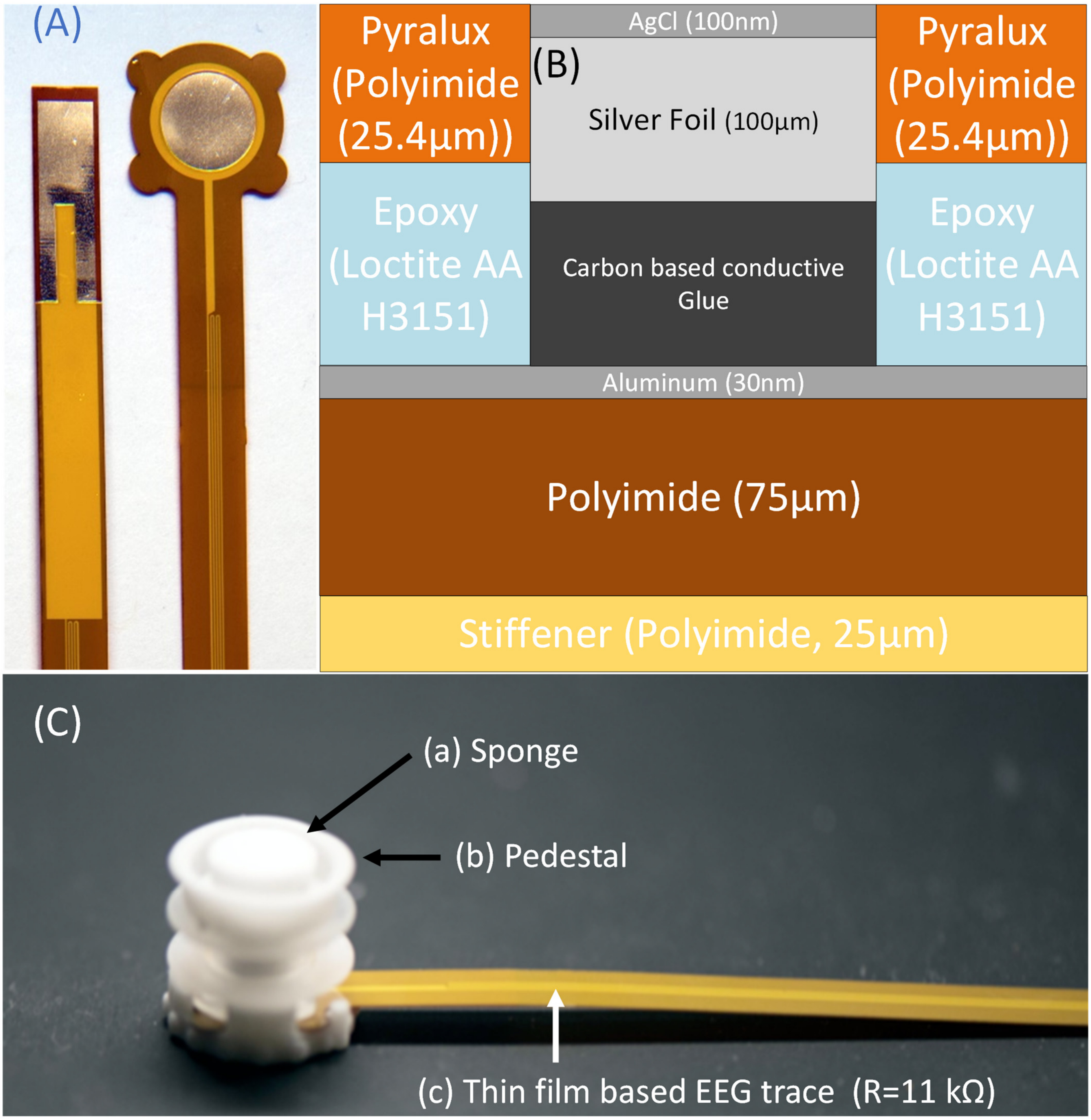

Figure S5: The Traces of the NeoNet. *(A) The detail image of electrodes. (B) The traces and electrodes layout in the old version 1 of the fabrication process. (C) An image of the traces with the electrodes and sponges.*

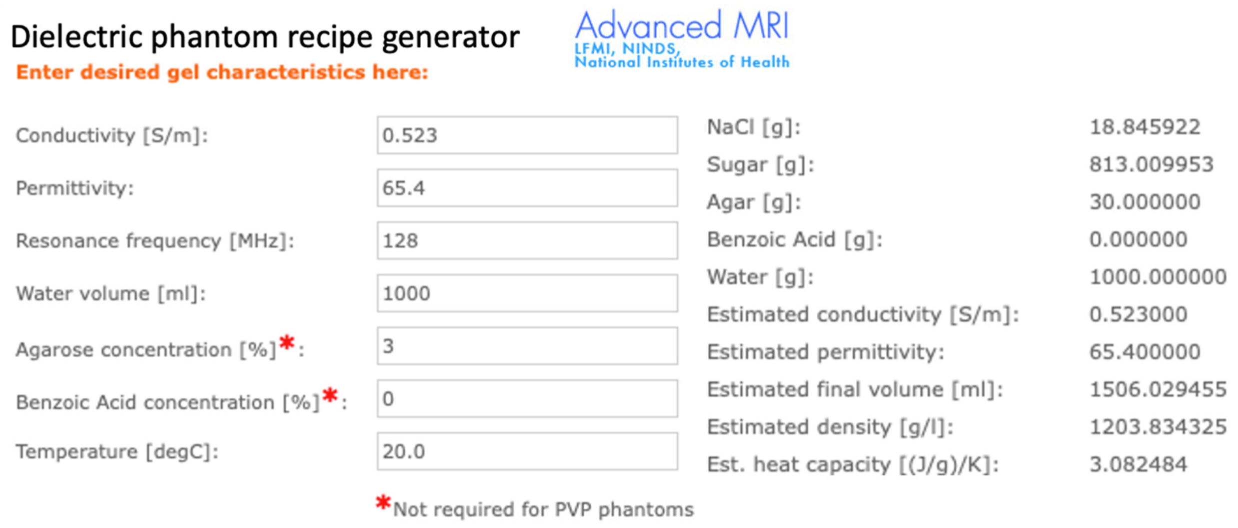

f=128 MHz	Recipe 1		Recipe 2		Saline (51.3 mM)	
3% Agar (Recipe 1&2)<br>DI water: 1L<br>GC Agar-Agar 340g	Sugar 500 g<br>NaCl 18.85 g		Sugar 425 g<br>NaCl 16.04 g		DI water: 1L<br>NaCl 3g	
	ε_r	σ (S/m)	ε_r	σ (S/m)	ε_r	σ (S/m)
Measured value	68.07	0.530	62.06	0.470	77.38	0.539
Estimated value	65.40	0.523	65.40	0.523	78.00	0.576
Differences	4.08 %	1.33%	3.47 %	14.72 %	0.79 %	6.37 %

Figure S6: The phantom recipe calculated from the dielectric phantom recipe generator [20]. The dielectric properties measurement results of two sample recipes at 128 MHz are displayed in the table. The measurement of the saline solution (51.3mM, NaCl 3g/L) was also compared to see the measurement accuracy.

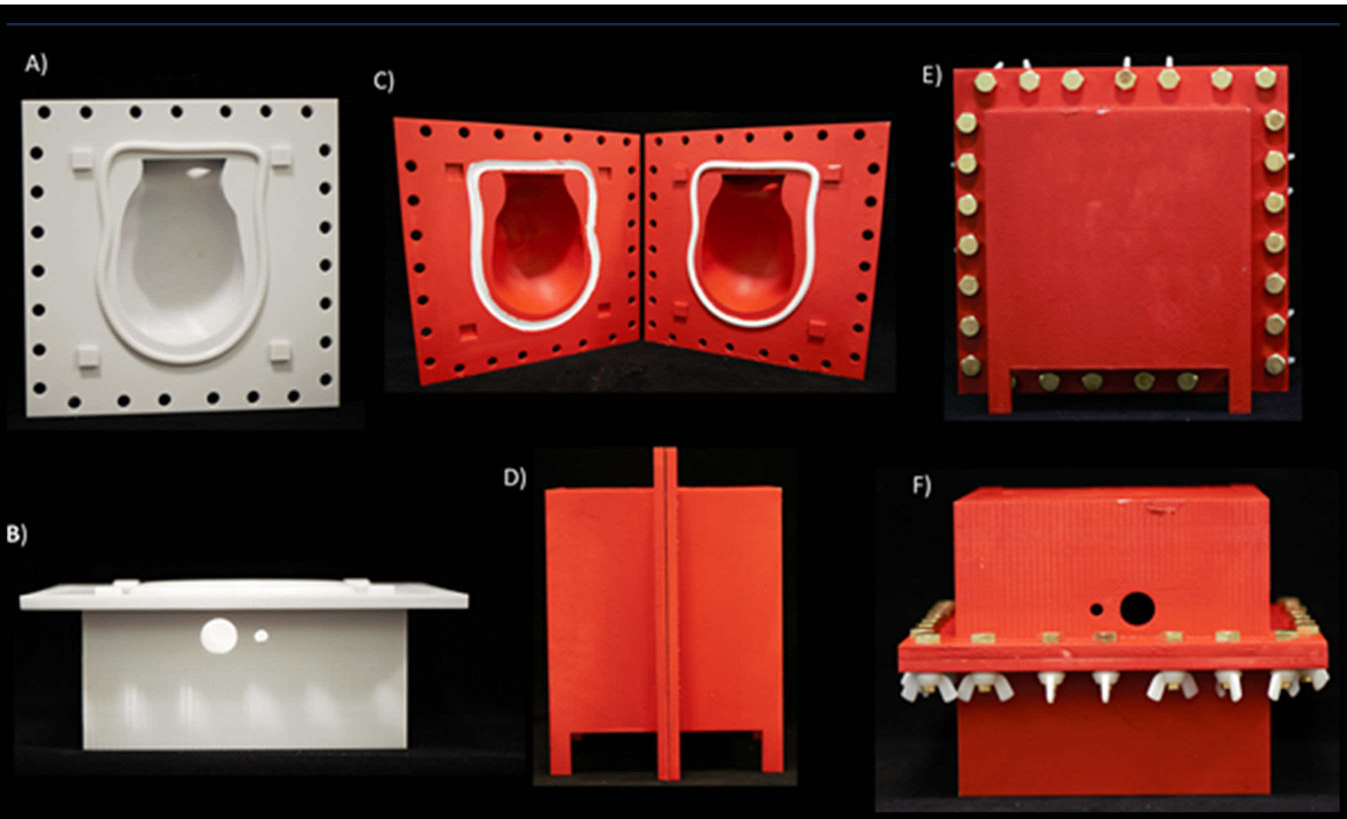

Figure S7: Phantom construction. A) After 3D printing with FDM (fused deposition molding) process, the polycarbonate phantom is white and is slightly porous. B) Two holes were created at the top of the phantom. One is for pouring the agar solution, while the other is to allow air to flow, effectively eliminating air pockets in the phantom. C) After 3-4 coats of red rubber spray paint, the phantom is ready to be put together. The rubber spray paint seals the phantom from agar solution leaking through the porous polycarbonate. The white channel was masked so that the two pieces may fit together seamlessly. D) The two pieces fit seamlessly together. E) Screws and wingnuts are attached to the phantom, creating a seal that will hold the two pieces together during the phantom fabrication process. F) There is enough space to fit a funnel into the larger hole for pouring.

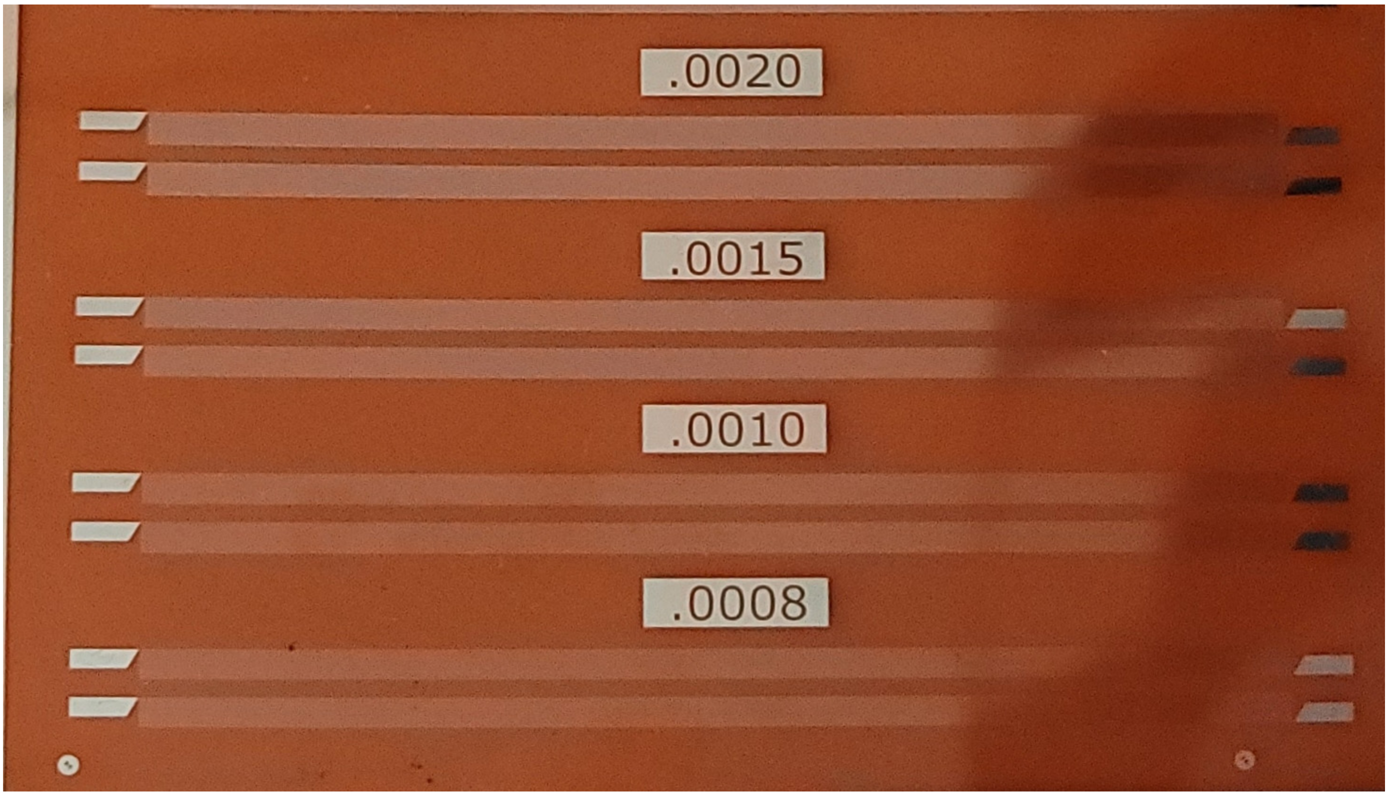

Figure S8: Coupon used for trace width testing.

References

1. Tzovla, A., et al., *20 Hypothermia-Treated Infants with Hie: Mri Findings in Relation to Short-Term Outcome.* Pediatric Research, 2010. **68**(1): p. 13-13.
2. Awal, M.A., et al., *EEG background features that predict outcome in term neonates with hypoxic ischaemic encephalopathy: A structured review.* Clin Neurophysiol, 2016. **127**(1): p. 285-296.
3. Bonmassar, G., et al., *Influence of EEG electrodes on simultaneous EEG/fMRI measurements.* Human Brain Mapping, 2001. **14**(2): p. 108-115.
4. Bonmassar, G., K. Fujimoto, and A.J. Golby, *PTFOS: flexible and absorbable intracranial electrodes for magnetic resonance imaging.* PLoS One, 2012. **7**(9): p. e41187.
5. De Vos, M., et al., *Automated artifact removal as preprocessing refines neonatal seizure detection.* Clin Neurophysiol, 2011. **122**(12): p. 2345-54.
6. Vidal, F., et al., *Linking EEG signals, brain functions and mental operations: Advantages of the Laplacian transformation.* Int J Psychophysiol, 2015. **97**(3): p. 221-32.
7. Kayser, J. and C.E. Tenke, *Issues and considerations for using the scalp surface Laplacian in EEG/ERP research: A tutorial review.* Int J Psychophysiol, 2015. **97**(3): p. 189-209.
8. Babiloni, F., et al., *Spatial enhancement of EEG data by surface Laplacian estimation: the use of magnetic resonance imaging-based head models.* Clin Neurophysiol, 2001. **112**(5): p. 724-7.
9. Babiloni, F., et al., *A high resolution EEG method based on the correction of the surface Laplacian estimate for the subject's variable scalp thickness.* Electroencephalogr Clin Neurophysiol, 1997. **103**(4): p. 486-92.
10. Song, J., et al., *EEG source localization: Sensor density and head surface coverage.* J Neurosci Methods, 2015. **256**: p. 9-21.
11. Bulteau, C., T. Otsuki, and O. Delalande, *Epilepsy surgery for hemispheric syndromes in infants: hemimegalencepahly and hemispheric cortical dysplasia.* Brain Dev, 2013. **35**(8): p. 742-7.
12. Wyllie, E., *Surgery for catastrophic localization-related epilepsy in infants.* Epilepsia, 1996. **37 Suppl 1**: p. S22-5.
13. Englot, D.J. and E.F. Chang, *Rates and predictors of seizure freedom in resective epilepsy surgery: an update.* Neurosurg Rev, 2014. **37**(3): p. 389-404; discussion 404-5.
14. Vanhatalo, S., M. Metsaranta, and S. Andersson, *High-fidelity recording of brain activity in the extremely preterm babies: feasibility study in the incubator.* Clin Neurophysiol, 2008. **119**(2): p. 439-45.
15. Stjerna, S., et al., *Preterm EEG: a multimodal neurophysiological protocol.* J Vis Exp, 2012(60).
16. Kwan, P. and M.J. Brodie, *Refractory epilepsy: a progressive, intractable but preventable condition?* Seizure, 2002. **11**(2): p. 77-84.
17. Kowalczyk, M.A., et al., *Dynamic analysis of fMRI activation during epileptic spikes can help identify the seizure origin.* Epilepsia, 2020. **61**(11): p. 2558-2571.
18. Omidvarnia, A., et al., *Dynamic coupling between fMRI local connectivity and interictal EEG in focal epilepsy: A wavelet analysis approach.* Hum Brain Mapp, 2017. **38**(11): p. 5356-5374.
19. Kowalczyk, M.A., et al., *Clinical benefit of presurgical EEG-fMRI in difficult-to-localize focal epilepsy: A single-institution retrospective review.* Epilepsia, 2020. **61**(1): p. 49-60.
20. Duan, Q., et al., *Characterization of a dielectric phantom for high-field magnetic resonance imaging applications.* Med Phys, 2014. **41**(10): p. 102303.
